# Genomic Insights into the Different Layers of Gene Regulation in Yeast

**DOI:** 10.4061/2011/989303

**Published:** 2011-11-22

**Authors:** José E. Pérez-Ortín, Daniel A. Medina, Antonio Jordán-Pla

**Affiliations:** Departamento de Bioquímica y Biología Molecular, Facultad de Biológicas, Universitat de València, C/Dr. Moliner 50, 46100 Burjassot, Spain

## Abstract

The model organism *Saccharomyces cerevisiae* has allowed the development of new functional genomics techniques devoted to the study of transcription in all its stages. With these techniques, it has been possible to find interesting new mechanisms to control gene expression that act at different levels and for different gene sets apart from the known cis-trans regulation in the transcription initiation step. Here we discuss a method developed in our laboratory, Genomic Run-On, and other new methods that have recently appeared with distinct technical features. A comparison between the datasets generated by them provides interesting genomic insights into the different layers of gene regulation in eukaryotes.

## 1. Functional Genomics Techniques as a Driving Force for Biology

In the 1980s, Sidney Brenner stated that, “…progress (*in biology*) depends on the interplay of techniques, discoveries and new ideas, probably in that order of decreasing importance” [1]. It is absolutely true that most scientific revolutions have appeared after technological developments which are, directly or indirectly, the bases for obtaining new kinds of data which, in turn, have led to the emergence of new ideas among contemporary scientists. This statement also holds true for the commonest case of nonrevolutionary developments. All new tools created by scientists or technicians are always followed by novel data which, in most cases, have led to new proposals, hypotheses, or theories.

Since genome sequencing projects began in the early 1990s, a new biology concept has started. This concept was not new, but the development of new technologies to sequence and analyze whole genomes provided such an amount of new data, that a new kind of biological science was born, the so-called “omics” [2]. Genomics and other omics sciences have made a real revolution of molecular biology itself. This is especially true because the preceding molecular biology was an especially reductionist science; that is, genes, proteins, and pathways were mainly analyzed and screened individually in an attempt to decipher each one in the most in-depth way possible. Obviously, although the search for relationships among genes, proteins, and pathways was also underway, all integrative approaches lacked the most important component to be fully developed: data. At the same time, molecular systems biology came into being after Jacob and Monod's operon model [3]. Although, it was restricted to a few genes, proteins, and pathways, it never attempted to check if proposed mathematical models were more or less common in cells, and it certainly never dreamed of building comprehensive models to explain the whole behavior of a living cell.

The sequencing of the first eukaryote, the yeast *Saccharomyces cerevisiae*, made it possible to develop a totally new field in biology: functional genomics [4]. Until that time, genomics was a science devoted to obtaining genome sequences and “in silico” analyses of them. Given the availability of a whole genome sequence for a model organism, for which a huge amount of biological information existed and because of the awesome power of yeast genetics, it was possible to develop totally new tools and specialized mutant collections in a relatively short time (see [2], for a review). It also provided the data for establishing molecular systems biology from an omics perspective [2]. The characteristic collaborative atmosphere of yeast genetics and a molecular biology community contributed to the rapid establishment of databanks (e.g., SGD or CYGD, [5, 6]), transnational projects [5], and strain repositories (e.g., Euroscarf), which are freely available for any interested scientist.

One of the most successful techniques in functional genomics has been microarray technology. Microarrays were fully developed by the mid-1990s, using mainly *S. cerevisiae* as a workhorse for many of the technological advances (reviewed in [7]). Different platforms have been created since 1997 for the whole yeast genome; for instance, glass cDNA microarrays [8] which have been the most widely used. Nylon macroarrays [9] were one of the first to be developed and are still a convenient alternative for specialized purposes [7]. Oligonucleotide arrays were developed by several laboratories and companies and are currently the most used alternative, especially the type known as tiling arrays, which cover the whole genome without leaving any gap in it. They have been used to discover totally unpredicted genes, noncanonical transcripts, either sense or antisense, as regards canonical genes [10] and to also locate the binding sites of many of the transcription-related proteins of this lower eukaryote [11].

Sidney Brenner's actual opinion does not correspond exactly to what people may think about his quoted sentence [1]. In fact, he has declared that this new emerging genomic approach is “low input, high throughput and no output biology” [12]. This opinion is widely extended among biologists because it seems that genomic techniques are just “fishing expeditions” in which there is no previous hypothesis to support them. Obviously this criticism is, at least in part, false. Each new genomic technique's own technical protocol is devoted to catching new kinds of fishes. Although the nature of these new fishes is not totally predicted in advance, it is rather obvious that there is a basal hypothesis in the technique's background: we are going to fish new, unknown specimens that will have special features that our new technique will be able to catch. A good example of this is the discovery of CUT (cryptic unstable transcripts) anywhere in the genome, but mainly in relation to canonical genes *loci* by means of tiling arrays and high-throughput sequencing (HTS) [13–15]. An additional corollary of this is the interpretation of the fishing expedition as not being a trivial question. Identifying the fishes and investigating the biological mechanisms that originate them are also ways of making science. In fact, the generation of new results is not only a natural consequence of the development of a new technique, but also a previous step to put forward a new hypothesis. For instance, the analysis of CUTs has brought about the discovery of new mechanisms of mRNA quality control and transcription termination [13].

In our lab, we study the whole gene transcription process using *S. cerevisiae* as a model system. RNAs, especially mRNAs, are unstable molecules. They are degraded by exo- and endonucleases, mostly in the cytoplasm [16]. The amount of mRNA (RA), therefore, is not just the result of transcription, but the equilibrium caused by transcription rates (TRs) and degradation rates (DRs). We realized that the genomic techniques available at the beginning of the 21st century were able to quantify RA, but that there were no techniques available to measure turnover rates. In 2002, Pat Brown's group developed a genomic technique to measure mRNA stabilities in yeast [17]. This technique was an upgrade of the well-established transcription shutoff protocols used for individual mRNA half-life studies [18]. These protocols cause stress in cells, which impedes the measurement of many mRNAs' half-lives involved in the stress response [19]. The cell's physiology is also affected: it is not necessarily true that the measured half-lives correspond to the real ones in nonperturbed cells [20]. For this reason, we developed a new protocol called Genomic Run-On (GRO), which is able to measure nascent transcription rates (TR) for all yeast genes and, at the same time, the mRNA amounts (RA) for them. In this way, mRNA stabilities can be determined from RA and TR in cases in which RA does not change, in steady-state conditions [21], and even during abrupt changes in RA after stress [20].

## 2. Genomic Run-On (GRO) for Yeast Cells. Features of the Nascent TR Dataset

**Figure 1:**
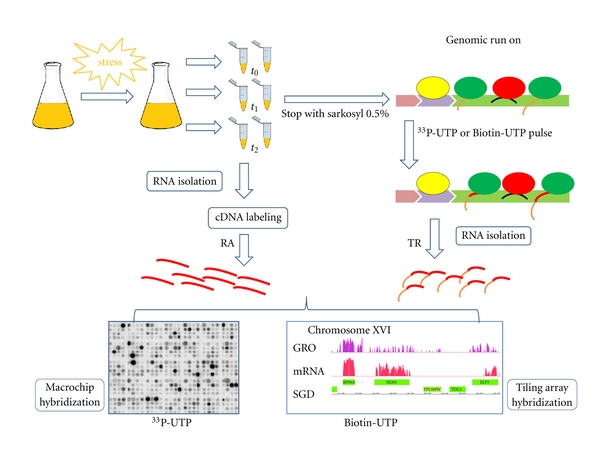
Outline of the GRO method for yeast cells. Cells grown to the desired condition are sampled in duplicate. One aliquot (arrow pointing rightwardly) is used for run-on labeling with either a radioactive precursor or 11-biotin-UTP. Total extracted RNA is used for macroarray or tiling microarray hybridization. The second cell's aliquot (arrow pointing downwardly) is frozen and used for RNA extraction and cDNA labeling, with either ^33^P-dCTP or biotin allonamide triphosphate, and hybridized in a new tiling microarray (BioGRO) or in the same macroarray (GRO) previously used after stripping. This method can be used for single point determination in different strains or can be easily adapted to time-point series after a stress or drug treatment, as shown. RNA pol II molecules are shown in different colors, indicating molecules before the elongation step (yellow), elongating (green), or backtracked (red). Labeled parts of mRNAs or cDNA are drawn in red. RA = mRNA amounts, TR = transcription.

The run-on method is a well-known procedure for detecting elongating RNA polymerases (RNA pols) in eukaryotic nuclei [22].  Figure 1 depicts the outline of the method. In most eukaryotes, it is necessary to isolate nuclei before doing the experiment [22]. In yeasts, however, whole cells can be directly used, which allows a physiological freezing of the actual transcription state in those organisms. The permeabilization of cells by means of a detergent (usually sarkosyl) provokes a sudden decrease of the NTP pools and stalls all elongating RNA pol. The detergent also disrupts the chromatin structure, thus avoiding any further initiation event. A subsequent pulse of externally added ribonucleotides, including labeled UTP (^33^P-UTP or derivatized-UTP), induces a death rattle of these RNA pols, which actively elongate. This *postmortem* elongation labels the RNA molecules with a natural sequence of about 200–300 nucleotides. Those RNA pols that were backtracked or did not elongate do not incorporate nucleotides (red and yellow ovals in Figure 1). Most nascent RNA becomes labeled in this way. This RNA is probably less than 1% of the total RNA in the cell. Because it is labeled, it is possible to use it as a hybridizable molecule in a dot blot or a DNA array or, alternatively, to purify it based on any unique property conferred by the UTP analog used. The latter could be used for high-throughput sequencing or for DNA microarray hybridization. The signal associated with a particular sequence (the probe in the DNA array) is a reflection of the RNA pol density in it. If we assume a constant speed for the RNA pol, then RNA pol density is proportional to its transcription rate. 

**Figure 2:**
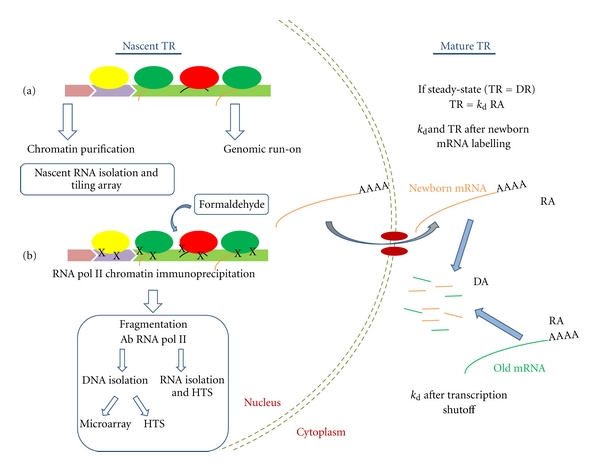
Outline of the different genomic techniques for TR and mRNA stability determination used in yeast. Determination of nascent TR in the nucleus is based on the detection of RNA polymerases or nascent RNAs. (a) Native chromatin can be purified and nascent RNA can be isolated. Then it is converted into cDNA and used for the tiling array analysis [32]. Alternatively, cells can be used for the GRO analysis (see Figure 1). (b) Cells can be fixed with formaldehyde and subjected to chromatin fragmentation and immunoprecipitation with RNA pol II antibodies. From this ternary complex, either DNA or RNA can be detached from polymerases and analyzed downstream. Recovered DNA is suitable for array hybridization (ChIP-Chip) [10, 33, 34] or HTS (ChIP-Seq) [35], whereas RNA can be converted into DNA and subjected to HTS [36]. The appearance of recently synthesized mRNA (newborn, orange) in the cytoplasm can be followed by thiouridine *in vivo* labeling, which is purified, quantified, and compared with the non labeled old mRNA (green). With this technique, it is possible to calculate mature TR and mRNA half-lives [37]. mRNA half-lives can also be calculated from a transcription shutoff experiment [38], from TR and RA data by assuming the steady state (*k*_d_ = TR/RA), or even for non-steady-state conditions [39]. DR: degradation rate. Other symbols are as in Figure 1.

In 2004, we developed [23] a genomic upgrade of the yeast run-on technique which we called “genomic run-on” (GRO). It is based on a subsequent hybridization of RNA extracted onto whole yeast genome nylon macroarrays [24]. This acronym has been used for a similar technique in human cells [25]. The fact that this technique is user friendly allows it to be used in many situations, such as the study of mutants, even when a metabolic fast change occurs. Indeed, 50 mL aliquots of yeast cells can be taken every two minutes if needed and processed very quickly in a few minutes. This allows to follow stress responses with a high resolution (see Figure 1; for a detailed experimental protocol; see [26]). In our protocol, another aliquot of cells is taken and frozen at the same time. This aliquot can be used for routine RNA purification (note that all RNAs are unlabeled in this case) and for subsequent standard mRNA amount determination after labeling as cDNA (Figure 1). Since both cell aliquots come from exactly the same time point of the culture, TR and RA values correspond to the average values of those parameters for a given cell population. The existence of experimentally determined TRs for all the genes of a given organism allowed us to compare the response profiles of each gene after an external change. We performed this kind of studies in the change from a glucose to a galactose medium [23] after oxidative stress [27], after osmotic stress [28] and after heat stress [29]. In all cases, genes cluster according to their response profiles by mostly following functional relatedness. This can be the direct result of the common regulation of those genes belonging to a same regulon by a transcription factor (TF). In fact, a meta-analysis done with our data by a different group showed that TR profiles were more suitable to predict functional relatedness than RA profiles [30]. The reason for this is quite obvious; nascent TR is the parameter directly affected by a TF. A change in RA can be the result of not only a change in TR, but also in mRNA stability (see Figure 2). Even the rate at which mRNA appeared in the cytoplasm (mature TR) could be less suitable for regulon finding because some posttranscriptional events, like mRNA export, can affect the mature TR profile. The effect of posttranscriptional changes blurs RA profiles because some mRNAs display different posttranscriptional behaviors. Therefore, the clustering of TR profiles seems to be the best tool to find transcriptional regulons. Although only formally demonstrated in *S. cerevisiae,* this statement seems to be reasonably extended to other organisms when methods to determine nascent TR become available. Nascent TR is also the best way to classify genes for active chromatin marks. It seems somewhat logical that the passage of RNA pol II molecules along the chromatin template is affected by particular nucleosomal organization. Because nascent TR, as determined by GRO, measures the actual elongation rate, what is actually affected by nucleosome positioning and readability should be a better predictor of the characteristic chromatin marks of active genes than mature TR which, as mentioned, considers other posttranscriptional steps. We have shown that this is precisely the case. When comparing the level of the different active chromatin features, such as H3-K_36_ trimethylation, or the presence of Esa1p or Gcn5p histone acetyltransferases, we found that the correlation with nascent TR (calculated by GRO) is better than when compared with the mature TR calculated from steady-state RA and mRNA stabilities [31].

**Figure 3:**
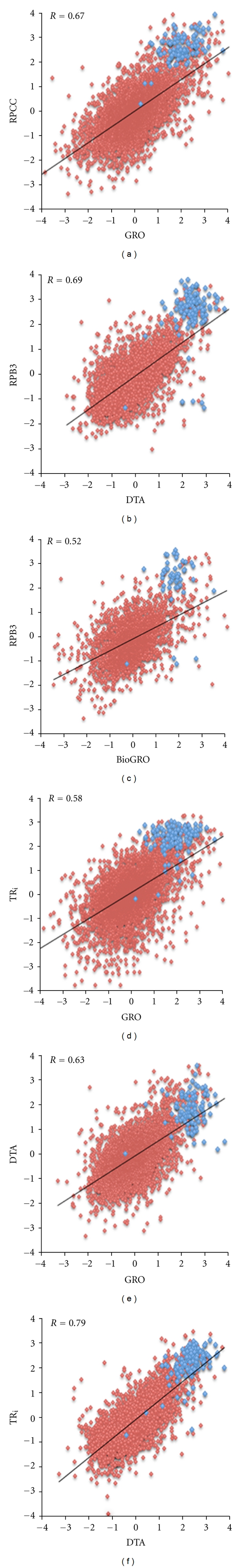
Comparison of the TR datasets obtained by different protocols. Available TR datasets were converted to log_2_ values and standardized by the Z-score. The tendency line for a linear correlation is shown. The *r* Pearson coefficient for the correlation is provided in the top left corner of each plot. In all cases, the RP genes (blue dots) distribution differs from the global gene distribution (red dots) according to the Wilcoxon text with a *P* value of <0.0001. GRO is the radioactive nascent TR dataset. BioGRO is an unpublished nascent TR dataset obtained with Biotin-UTP and tiling arrays (Jordán-Pla et al, unpublished). RPCC is the RNA pol II chromatin immunoprecipitation performed by us [31]. RPB3 is the RNA pol II chromatin immunoprecipitation performed by Mayer et al. [33] using an anti-RPB3 antibody.

The GRO protocol has allowed us to obtain a whole TR dataset for an organism for the first time [31]. Analyzing the dataset provided a number of surprises: 90% of yeast genes show TRs between 2 and 30 molecules/h, with a median of 7 molecules/h. This corresponds to 0.078 RNA pol II molecules/kb or 0.1 molecules/gene. As 25% of transcription corresponds to 5% of most transcribed genes, the distribution of RNA pol II molecules in a snapshot of an actively growing yeast cell is mostly like a desert: only 14% of genes have any actively transcribing RNA pol II molecule. Transcription onto canonical genes does not seem to be a common feature of the yeast genome in spite of the high number of RNA pol II molecules present in a cell (20000–30000) since only around 700–1400 would transcribe genes that encode proteins at a given time. One possibility is that part of these molecules is unable to transcribe, and that the amount of CTD-phosphorylated molecules (12000, according to [40]) suggests that they can be transcribing in other regions outside the canonical genes (see below), or perhaps that the mRNA molecules reaching the cytoplasm are merely a fraction of the nascent ones. The most transcribed genes are those that code for histones, which reach about 206 mRNAs/hour during the S phase. These results are rather similar to those obtained by a different technique (dynamic transcriptome analysis (DTA) in [37], see below) which measures mature TR. The similarity between total nascent and mature TRs, however, does not mean genuine equality because the absolute units were obtained by normalization in both cases against the amount of mRNAs per cell. In any case, the similar medians and distributions, and the high correlation (r Pearson 0.63; see Figure 3) obtained, favor the quality of both datasets. 

TR is not dependent on G+C content, but decreases for increasingly long genes [31]. This result was expected because of the probability of RNA pol II failure increasing with transcription unit length [41, 42]. The slope of this bias is greater for the GRO dataset than for the other TR evaluation method based on RNA pol II crosslinking to the gene (RNA pol II chip-on-ChIP: RPCC; see below). Moreover, the GRO curve shows a change in tendency for those genes whose ORFs are longer than 3 kb (see [31], Figure  S1). This effect can be caused by the macroarrays used for both RPCC and GRO in which any gene whose ORF length is longer than 3 kb is represented by a probe covering only the last 1 kb downstream in the ORF, whereas the rest of the genes (<3 kb) are represented by the whole of the ORF. As the GRO method labels the elongating mRNA by extending it 200-300 nt downstream, it is expected to bias the label to the 3′ part of the genes. This would lead to an increased average label in the genes as their length shortens and the relative influence of their 3′ part increases. It also predicts a sudden increase for the genes represented by probes that cover only their last 1 kb of the ORF. With this in mind, we conclude that there is a general bias in the GRO which increases the calculated TR inversely with the ORF (i.e., probe) length. Additionally, there is an artifact for ORFs that are longer than 3 kb due to the use of 1 kb probes from their 3′ end. This gene-/probe-dependent effect was corrected by using the RPCC data for the lowest normalization of the GRO data. Our TR dataset has, therefore, been corrected for this artifact. 

Another potential artifact of GRO (and other nascent TR methods) is the potential effect of cryptic transcription. Since GRO labels any elongating RNA polymerase, those RNA pol II molecules, that elongate anywhere inside the genome regions contained in probes (ORFs), are labeled regardless of making “canonical” transcripts or cryptic transcripts. In the original GRO method [23], the macroarrays used contain dsDNA probes. Because of this, both sense and antisense transcriptions will be summed. The existence of a vast number of cryptic transcripts has been demonstrated in many organisms, including yeast (see [42], for a recent review) in which two types have been defined: cryptic unstable transcripts (CUTs), which are only detectable in the absence of nuclear exosome activity and stable uncharacterized transcripts (SUTs) [14]. Some authors argue that cryptic transcription can be responsible for the differences observed in the genes' response to stress situations when comparing mRNA data and TR data [15, 43]. This contrasts with the recent unveiling of experimental evidence diminishing the possible quantitative contribution of antisense transcripts to the RNA pool when compared to their stable sense transcript counterparts in the bidirectional promoter's context [36, 42]. Moreover, we analyzed the different cryptic transcription datasets published and we observed that they are quite different, with very little overlap, and that the technique used to find them vastly affects the type (sense or antisense) of the cryptic transcripts found (García-Martínez et al., submitted). Thus, it seems that most yeast genes have cryptic transcripts, but mainly in a very low proportion (discussed in [42]). Thus, although we agree that cryptic transcription is a real contributor of nascent TR data, we believe that nascent TR reflects mainly “canonical transcription” for most genes.

## 3. Alternative Methods for Evaluating Transcription Rates and mRNA Degradation Rates

Thus the GRO technique allowed the comparison of the rate at which each mRNA is produced and its amount in the cell. This idea has been used quite recently by other authors, who have followed different TR evaluation methods (see below) both in yeast [37] and higher eukaryotic cells [44]. In all these studies, mRNA stabilities have also been calculated. The possibility of calculating mRNA half-lives indirectly from TR and RA is based on the aforementioned equilibrium between TR and DRs. If they are equal, then RA is constant over time. This is known as a steady-state situation, which mainly occurs for most mRNAs. For instance, we demonstrated that when yeast is grown in a flask (a batch culture), most of the exponential growth phase maintains the steady state for most mRNAs [21]. Although some mRNAs change slightly after several hours, the steady-state condition can be a good approximation to describe the mRNA pool. It is likely that the steady state is also true for the stationary phase or in chemostat cultures [45]. As DR follows a first-order kinetic law, it is proportional to RA and to a degradation constant (*k*_d_). The GRO protocol calculates RA and TR for all the yeast genes. Because DR = TR, it is also possible to calculate *k*_d_ for them all. *k*_d_ has time^−1^ units and the reverse meaning of the half-life (*k*_d_ = ln2/half-life). When no steady-state situation occurs, again the chemical kinetic laws can be used to develop an equation in which the successive TR and RA time points are employed to infer *k*_d_ using the simplification, whose changes in both are lineal between the successive time points [20]. Although the mathematical computation increases the experimental associated error, this approach has allowed us to calculate *k*_d_ variations for those groups of genes with common RA and TR profiles during fast stress responses [27–29]. In all the individual genes tested, the *k*_d_ calculated at different times during the stress response qualitatively coincided with the experimentally determined one using Tet-off promoters. 

In the last few years, other techniques apart from GRO have been developed to study TRs and mRNA stabilities in yeast and in other organisms at the genomic level. In all cases, they use recently developed, higher resolution methods, such as tiling arrays or high-throughput sequencing (HTS), which provide deeper insights into the transcription process than when using classical DNA arrays. For TR determination, most methods focus on nascent TR (Figure 2). The classical approach to unveil the dynamics of the transcriptional process at the TR level relies on the generation of RNA pol II density landscapes to precisely map where RNA pol sit in the whole genome, regardless of transcriptional states (active, paused, backtracked, etc.; see Figure 2). This has been achieved with chromatin immunoprecipitation methods coupled with microarray analysis, ChIP-chip [33, 37, 43] or HTS, ChIP-Seq [35]. With these high-throughput methods, a plethora of different occupancy profiles for RNA polymerase II and its different phosphorylation forms are now publicly accessible. However, ChIP techniques cannot circumvent the fact that the presence of polymerase in a region should not be directly assumed as actual transcription because of there being nonelongating polymerases and because ChIP-associated techniques cannot discriminate the sense/antisense transcripts (see above). A recent variation of ChIP techniques has been able to partially skirt this drawback (Figure 2). By isolating and deep sequencing the nascent transcript associated with immunoprecipitated RNA polymerases (NET-Seq, [36]), both problems are avoided. Nascent mRNAs have been alternatively purified by chromatin fractionation approaches [32], thus providing a more direct measure of TR. The nonradioactive variants of GRO, coupled with tiling array analysis (BioGRO, Jordán-Pla et al., unpublished), or HTS (GRO-Seq), have been successfully applied to dissect the regulatory circuitry of yeast and human [25] cells. These high-resolution GRO techniques are beneficial because they can discriminate between active and nonactive transcription elongation states and can also detect any type of noncoding unstable transcript RNA polymerases generated in both the sense and antisense orientations, for which accumulating evidence shows that they play a crucial role in the regulation of gene expression [14], thus broadening our knowledge and understanding of gene regulation dynamics. 

Mature TRs determine the rate of appearance of newborn mRNA in the cytoplasm (Figure 2, right). *In vivo* metabolic labeling of transcripts, with uracil or uridine analogs pulses, subsequent mRNA isolation, fractionation between labeled (newborn) and nonlabeled (old) mRNA and analysis, is an essentially nonperturbing system which provides us with a way of measuring mature TR directly [46]. The recently developed DTA methodology in yeast [37] and mammalian cells [44] focuses on newborn mature polyadenylated transcripts and uses a metabolic time lapse of variable extent (6 minutes in yeast). DTA technology is currently the only technology available that is able to measure mature TR in yeast experimentally. It can also determine mRNA half-lives at the same time. The technique is, however, time consuming and assumes that TR and DR are constant during the time lapse used. It also leaves an important bulk of non coding transcripts outside the frame [14]. Mature TR can also be calculated from RA and mRNA stability datasets indirectly (indirect TR, TRi = *k*_d_ RA) by assuming a steady state [47]. Alternatively, mRNA half-lives (*k*_d_) can be calculated indirectly from experimental mature TR and RA datasets using the same equation or directly using the previously commented transcription shutoff methods [17, 19, 21].

The comparison of all existing yeast TR datasets [31, 33, 37, 47] (Jordán-Pla et al., unpublished) with each other and with a standardized RA dataset [31] has revealed that they all correlate quite well (Figure 3). Those corresponding to nascent TR correlate better to each other. This is also true for those corresponding to the mature TR (DTA, [37]; TRi, [47]). The last ones better correlate with the RA dataset. These results are logical because nascent mRNAs should be processed and exported to the cytoplasm where mature TR is measured. Moreover, nascent TR can be affected by cryptic transcription and mature TR cannot. The specific distribution of ribosomal protein (RP) genes (blue dots) is biased in all the comparisons shown. The meaning of this behavior is commented below.

## 4. Ribosomal Protein Genes: A Special Case of RNA Pol II Transcription

Ribosomal proteins in yeast are coded by a set of 137 genes. They are, perhaps, the most statistically significant group that clusters together in many of the genomic analyses done in yeast [23, 34]. This can be due to the fact that this group is more coordinated and/or more numerous than other regulons which are less coordinated (e.g., ribosome biogenesis regulon, RiBi, and *∼*200 genes), less abundant (Gal regulon, 7 genes), or both. Because translation is the most costly synthesis process for the cell [48], and as the ribosome is composed of stoichiometric amounts of RP, both the control and coordination of these genes is very strict. In yeast, they are mainly regulated at the transcriptional level [48], involving several TFs (see [49, 50], for a detailed updated review). RP mRNAs are among the most abundant in the cell. They have also been traditionally considered the most transcribed ones, representing as much as 50% of transcribing events [48]. Our results, however, quantify the overall TR of those genes as only 8.5% of total RNA pol II TR [31]. As previous estimations were based on indirect evaluations of RP transcription rates [48], we considered it merely a miscalculation. However, we now think that the main mistake seems to lie in the use of very few examples of RP mRNA half-lives at the time of the proposal. We recalculate now the total TR for RP genes (indirect TR or TRi) using all the available datasets of mRNA half-lives [17, 19] and RA datasets [31, 37]. In all cases, these genes represent less than 27% of total RNA pol II transcription in yeast. Therefore, the original 50% was clearly miscalculated. On the other hand, the plots of direct estimations of TR versus indirect ones (Figures 3(d) and 3(f)) show an overestimation of TRi values. The contribution of RP TR to the total can also be currently calculated from mature TR DTA data [37] to represent 16% of total TR. The differences with the previous 27% may be due to either a mistake in TRi or to a specific bias in DTA and GRO techniques for these genes. However, we think that the main reason is that transcription shutoff methods underestimate RP mRNA half-lives as they can provoke destabilization caused by stress. We have shown that this phenomenon does occur in all the stress responses we have analyzed to date (see below). In fact, RP mRNAs are 50% more stable than average in the DTA dataset [37] instead of merely coming close to it in transcription shutoff experiments [17, 19], or being less stable than average, as stated in Warner's review [48]. 

On the other hand, the bias observed in the RP nascent TRs measured by GRO, as mentioned above, may provide relevant insights into the elongation mechanism of these genes. We have seen that RP genes show a high percentage of nondetectable RNA pol II by GRO [34]. In exponential growth in glucose, they have about 36% more RNA pol II molecules present in the coding region of the RP, which are unable to do a run-on, more than the genome average (see Figures 3(a)–3(c)). We concluded that this reflects how the transcription of these genes involves a higher percentage of non elongating (probably backtracked) RNA pol II [31, 34]. This bias toward nonactive RNA pol II is also seen when comparing the results from mature TR (DTA technique, [37], Figure 3(e)) and RNA pol II immunoprecipitation [33], which reinforces our previous calculations. In this case, excess lies at about 31%, meaning that in RP genes, there is more RNA pol II present in those genes that do not produce mature mRNA in the cytoplasm than for the average gene. From the difference between the 36% and the 31% excess when using GRO or DTA in the comparison, or by directly comparing the DTA and GRO datasets (see Figure 3(e)), we conclude that between 15% and 40% of the RNA pol II molecules which are not labeled during the run-on are unable to resume elongation and to produce mature mRNA. This is perhaps because they are trapped in some step of the backtracking process [51]. The rest of the non labeled molecules (60–85%) would thus represent the efficiency of the backtracking process in recovering paused RNA pol II molecules, at least, for this group of genes.

What is the reason for the special abundance of non elongating RNA pol II molecules over RP genes? We hypothesized that a special chromatin structure of those genes could be responsible. In fact, TF Rap1p has a known chromatin-organizing activity (reviewed in [49]), which is predicted or demonstrated to activate 127 of the RP genes. However, this factor plays another, apparently opposed, role in telomeres: it organizes repressive subtelomeric chromatin [49]. Some years ago, it was shown that repressor activity resides in the C-terminal part of the protein, the *sil* domain, that a mutated version of Rap1 lacking it, *rap1-sil,* derepresses the genes within 50 kb from the telomere, and that it has increased levels of RP mRNAs [52]. We found that the excess of non elongating RNA pol II molecules disappeared in the *rap1-sil* mutant, which also occurs in a *tpk* single mutant [34]. Tpk1,2,3 are the alternative catalytic subunits of protein kinase A (PKA), which controls signaling from glucose [53]. Our model reveals that Rap1 not only recruits RNA pol II to the RP promoters, but it also organizes a partially repressed chromatin, which hinders RNA pol elongation, thus leading to pauses and arrest. This difficulty occurs at the beginning of the transcribed region [34] and could be an additional control mechanism for the regulation of these important genes. This mechanism would act only at the highest transcription rates (during exponential growth in glucose-rich media) as a way to accumulate truly elongating RNA pol II molecules which, due to this mechanism, slow down in the first part of the gene. After passing the control, these molecules increase their velocity and become sufficiently separated to avoid collisions. A similar elongation control has been proposed for ribosome translation [54] and has been mathematically demonstrated to increase the speed of RNA polymerases and to reduce noise (cell-to-cell variation, [55]), which is a typical, convenient feature of RP genes [56].

## 5. The Relative Importance of TR and DR in Controlling mRNA Amounts

At any time, RA is the result of the balance of TR and DR. When RA has to be changed, it is theoretically possible to act only on one side of the equilibrium, or on both sides. Traditionally, most studies on RA conducted at either the single gene level or the genomic level implicitly assumed that changes were due to only TR changes. A given gene is induced because a TF binds a promoter and attracts RNA pol II to transcribe it (by increasing TR) which, in turn, increases RA. Thus, RA profiles were considered to be a mere consequence of TR profiles. A few years ago, some authors started to pay attention to the potential effect of DR in the gene expression. Theoretically, the same effect on RA can be obtained by a change in DR rather than by an inverse change in TR. The mRNA half-lives determined by transcription shutoff methods were found to be very different between mRNAs and organisms [16]. More importantly, they were discovered to change for a given mRNA in different physiological situations [16]. When genomics strategies appeared [17], it was seen that the mRNAs belonging to the same pathways or functions tend to have similar half-lives, suggesting that regulons also exist at the mRNA stability level [57]. The trans factors acting in these posttranscriptional regulons were found to be mainly RNA-binding proteins (RBPs), which were relatively selective in the mRNA population because of their sequence specificity [58, 59].

As the GRO technique is able to indirectly determine mRNA stabilities in steady-state situations, as explained above, our first experiment [23] enabled us to reveal that those genes belonging to functionally related groups behave coordinately in DR. This was the first formal demonstration of the existence of post-transcriptional regulons. Similar studies in other organisms arrived at comparable conclusions, although not a whole genome-scale level (e.g., [38, 60]). In that first experiment, the times selected after changing cells from a glucose to a galactose medium were separated by hours, and the steady-state conditions can apply to each of them. Fast responses, typical of stress situations or sudden changes, did not meet the steady-state conditions. For such cases, we developed an algorithm, as previously described. With it, we have been able to determine approximate *k*_*d*_ profiles in response to different stresses for most genes and to verify the hypothesis of the influence of DR changes on RA profiles. Our studies [27–29] and those of others [61, 62] reveal that many genes have undergone changes in DR during stress responses. Many other genes, however, do not change their mRNA stability substantially. In line with this, interesting differences have been noted between various stresses [61], which probably depend on stress intensity. A good number of genes respond by slightly decreasing their RA level transiently to recover after several minutes. These genes tend to have flat *k*_*d*_ profiles, meaning they result from a mere transient decrease in TR. Other genes that respond to stress by lowering their RA are the RP and RiBi genes. In all cases, we have seen that the mRNAs of all these genes showed a transient destabilization, which can be accompanied by different degrees of TR decrease. These results indicate that DR can be used to reduce mRNA levels and, as explained later, to also speed up this reduction. It is interesting to note that some genes, which do not exhibit coordinated behavior at the TR level in some instances, actually display coordinated behavior with mRNA stability. This is the case of mitochondrial RP genes which cluster in the mRNA stability analysis, but not in the TR analysis, during the shift from a glucose to a galactose medium [23]. These genes do not present obvious regulatory elements in their promoters, but a Puf3 element in their 3′UTR has been demonstrated to coordinate their stability after changes in respiratory behavior [63]. Thus, our study was able to show that, for some specific gene categories, coordination takes place at the posttranscriptional level and that DR is the main player in shaping a response.

Those genes that positively respond to stress by increasing TR also show interesting changes in DR. For instance after osmotic stress, many genes present increased RA by increasing their TR and decreasing their DR (increase in mRNA stability) for several minutes [28]. A similar observation was reported by other authors using a different method to determine mRNA stabilities [61, 62] in the oxidative stress response. Thus after some minutes of osmotic stress, these genes reverse the change in DR in parallel to transcription shut-off [28]. This effect has been interpreted as the DR change which precedes changes in RA [62]. Other authors postulate that the changes in DR in both yeast [61] and mammalian [44] cells contribute to sharp response peaks. In mammalian cells with substantially longer mRNA half-lives, the contribution of DR changes to speed up the increases and decreases in mRNA levels is probably more important than in yeast. Nevertheless, the short yeast generation time (~100 minutes) and the need for faster, more economically adjusted responses to environmental situations than for mammalian cells mean that it is also necessary to use changes in mRNA stability to sharpen RA peaks in order to restrict energy expenditure while they take place [20]. The width of the TR response peaks seen in all the stress-activated or repressed genes is about 15–30 minutes, which is somewhat narrower than the RA peaks in our experiments and in those performed by others [64]. It has to be considered that the results obtained in experiments using about 10^9^cells are the average of all the possible individual states for a given cell. The difference in RA between individual cells is known as “transcriptional noise”. It has been determined to considerably differ for two kinds of genes: the TATA-less genes, with nucleosome-depleted constitutive promoters, for example, RP genes [49], which are less noisy [56] than the inducible genes with a TATA box at their SAGA-dependent promoters, known to be transcribed in “bursts” of several consecutive mRNAs separated in time [65], and thus in a much noisier manner [56]. Stress-activated genes belong to this last group. Thus, it is conceivable that the relatively sharp TR peaks result from much sharper peaks in individual cells in which a single (or very few) transcription burst occurs at their single gene locus during a stress response. At the molecular level, this would correspond to a switch from off to on in the promoter chromatin structure. The consequence of a sharp TR peak in an RA peak would be sharp only if mRNA has very low stability (see [20]). Therefore for mRNAs with half-lives longer than 10–15 min, it is necessary to unstabilize them if a fast return to the original mRNA level is needed. At the molecular level, this also assumes a binary switch in a given cell represented by a change in the affinity of an RBP to its cognate 3′-UTR mRNA element [59]. In this case, however, the existence of multiple mRNA molecules per cell probably makes the “degradation burst” less acute. 

 All these results suggest the importance of DR in controlling mRNA levels during transcriptional responses. Reciprocally important quantitative analyses of TR and DR in shaping RA have shown that despite being theoretically equivalents, TR and DR do not seem to play the same role in determining the amount of mRNA in stress responses or at steady-state conditions. It is interesting to note that when comparing the different datasets for TR, RA, or mRNA half-lives, in all cases TR and RA show a positive significant correlation, which mRNA half-lives never do (e.g., stable mRNAs are not the most abundant, and unstable mRNAs are not the least abundant); indeed, they even show slightly negative correlations with RA and TR in yeast [37, 39] or mammalian [66] cells. When measuring the number of genes whose response profiles are significantly affected by DR changes, we [23, 27, 29] and other authors [44, 66], found a large majority of genes in which TR changes are the main determinant of RA profiles. Thus, it seems that DR is not used for the quantitative control of most mRNA levels in the majority of situations, but for classifying them into rapid or less-rapid response genes according to the stability of their mRNA [20, 44]. Many mRNAs have a relatively constant DR. Nevertheless, some special gene categories, such as stress-induced or RP genes in stress responses in yeast (see above), mitochondria-related genes during metabolic reprogramming from glucose to galactose in yeast [23], or inflammatory and immune signaling genes in dendritic cells [44], have a highly variable DR because of the cis elements in their 3′-UTRs targets of specialized RBPs [29, 58, 59]. In some instances in which growth stops, like the stationary phase [67], after a change from a glucose to a galactose medium in yeast or after strong stress, a general change in DR is observed [23, 68]. This situation probably relates more to a general change in DR machinery and/or p-body organization and can overlap with particular responses.

The corollary of this scheme is that each particular group of genes has a TR through their promoter organization, and a DR through their particular 3′-UTR sequences, which are subjected to transcriptional (regulons) and posttranscriptional (posttranscriptional regulons) regulation [58]. Both should evolve coordinately to achieve a common expression strategy (CES) for the group of genes. We analyzed the existence of CES not only for transcriptional regulation, but also for translational regulation [39]. We found that our hypothesis was true: each analyzed functional gene category had a statistically significant CES for both transcription and translation. Genes whose protein products belong to large stable stoichiometric complexes, such as the cytosolic ribosome, the nucleosome, the proteasome, and many others, present characteristic profiles with relative unstable mRNAs and proteins, as well as relatively high transcription and translation rates. This probably reflects the need for fast changes in both mRNA and proteins in some instances to coordinate the amounts of subunits during a cell's life. Energy pathways genes, however, have more equilibrated profiles. A similar study in mammalian cells was done to find that CES also exists, but not quite the same as in yeast. For instance, in this case, RPs show very stable mRNAs, and proteins are very much like energy pathways genes [66]. These analyses reveal that, as expected, translation (translation rate and protein stability) is also a layer for gene regulation in all eukaryotes. From our study of yeast CES profiles, we conclude that the transcriptional layer is quantitatively more important for gene regulation than the translational one. However, Schwanhäusser et al. [66] conclude that the layer of translation is more important for determining the abundance of proteins in mouse fibroblasts. Hence, it seems that every life style needs particular gene expression strategies.

## 6. Future Trends

The similarities and differences observed between yeast and mammalian cells in organizing gene regulation indicate that the multiple layers used by eukaryotes provide a flexible network upon which every gene class can find its best strategy. Furthermore, this fitting can evolve according to the organism's requirements.

Variability in the single-cell gene expression in both microorganisms and tissue cells indicates that genomic transcription measurements should be complemented by techniques designed to quantify the gene expression at the single cell level [65, 69] and on the genomic scale. 

Given the adjustment required between TR and DR (and also translation), it seems that cells have to contain mechanisms that allow cross-talk between mRNA transcription and degradation at both the single mRNA level (by coordinating transcription from a promoter with the fate of the mRNAs producing that) and the general level to coordinate the regulons and posttranscriptional regulons composed of several different gene species. The recent publications by M. Choder's group [70] and others [71] reveal that such mechanisms do exist.

Finally, it is worth mentioning that most of the results reviewed herein would not have been possible without comparing large datasets because the general tendencies and the differences between genes categories are only significant if we look at the whole genome at the same time. Therefore, we consider that although anyone is free to believe that high throughput genomic technologies may be of “low input”, nowadays it comes over quite clearly that they actually provide some output to molecular biology.
